# Ischemic axonal injury up-regulates MARK4 in cortical neurons and primes tau phosphorylation and aggregation

**DOI:** 10.1186/s40478-019-0783-6

**Published:** 2019-08-20

**Authors:** Eric Y. Hayden, Jennifer Putman, Stefanie Nunez, Woo Shik Shin, Mandavi Oberoi, Malena Charreton, Suman Dutta, Zizheng Li, Yutaro Komuro, Mary Teena Joy, Gal Bitan, Allan MacKenzie-Graham, Lin Jiang, Jason D. Hinman

**Affiliations:** 1 Department of Neurology, David Geffen School of Medicine, University of California, Los Angeles, 635 Charles E. Young Dr. South, Rm 415, Los Angeles, CA 90095 USA; 2 Brain Research Institute, University of California, Los Angeles, Los Angeles, USA; 3 Molecular Biology Institute, University of California, Los Angeles, Los Angeles, USA

**Keywords:** Stroke, Ischemia, Subcortical stroke, White matter, Tau, Mark4

## Abstract

Ischemic injury to white matter tracts is increasingly recognized to play a key role in age-related cognitive decline, vascular dementia, and Alzheimer’s disease. Knowledge of the effects of ischemic axonal injury on cortical neurons is limited yet critical to identifying molecular pathways that link neurodegeneration and ischemia. Using a mouse model of subcortical white matter ischemic injury coupled with retrograde neuronal tracing, we employed magnetic affinity cell sorting with fluorescence-activated cell sorting to capture layer-specific cortical neurons and performed RNA-sequencing. With this approach, we identified a role for microtubule reorganization within stroke-injured neurons acting through the regulation of tau. We find that subcortical stroke-injured Layer 5 cortical neurons up-regulate the microtubule affinity-regulating kinase, *Mark4*, in response to axonal injury. Stroke-induced up-regulation of Mark4 is associated with selective remodeling of the apical dendrite after stroke and the phosphorylation of tau in vivo. In a cell-based tau biosensor assay, Mark4 promotes the aggregation of human tau in vitro. Increased expression of Mark4 after ischemic axonal injury in deep layer cortical neurons provides new evidence for synergism between axonal and neurodegenerative pathologies by priming of tau phosphorylation and aggregation.

**Electronic supplementary material:**

The online version of this article (10.1186/s40478-019-0783-6) contains supplementary material, which is available to authorized users.

## Introduction

Understanding the consequences of axonal injury on cortical neurons, particularly subcortically projecting Layer 5 cortical neurons, has wide-reaching implications in a variety of neurologic diseases. Importantly, subcortical ischemic axonal injury in the form of stroke is both common [18] and progressive [13] and contributes to the development of cognitive impairment and Alzheimer’s disease (AD) [16]. Though axonal injury within the white matter is associated with both sporadic and familial AD [26, 27], to date, no studies have suggested a molecular link between axonal injury and neurodegeneration. In part, the lack of models featuring isolated axonal injury within subcortical structures coupled with the complexity of axonal projections from cortical neurons poses a challenge to identifying molecular pathways that drive the selective neuronal loss common to both white matter lesions [12] and AD [40].

Models of axonal transection and crush injury in the peripheral, cranial, and optic nerves have provided tremendous insight into the molecular response of neurons to distal axonal injury, including the identification of dual leucine zipper kinase (DLK), Jun kinase and other molecular pathways [15]. Increasingly, these pathways are being studied outside the context of peripheral nerve regrowth and explored in models of neurodegeneration. Loss of DLK signaling can protect against axonal degeneration and neuronal loss in models of AD [25]. Jun kinase is implicated in neurodegeneration following injury and can directly phosphorylate the microtubule associated protein tau and promote the formation of neurofibrillary tangles that drive neurodegeneration in AD [51]. Despite these convergent mechanisms, evidence linking ischemic axonal injury in the white matter and molecular pathways that drive AD-related neurodegenerative phenomena is lacking.

In this study, we utilized a mouse model of focal ischemia within subcortical white matter that leads to axonal loss [37, 48] and selective neuronal injury within the overlying cortex [19]. We combined this model with layer-specific cortical neuron cell capture after stroke to identify a role for microtubule reorganization in stroke-injured cortical neurons. Using tissue clearing, we show that cytoskeletal reorganization in cortical neurons damaged by subcortical stroke selectively reduces apical dendrite length. RNA-sequencing of stroke-injured layer 5 cortical neurons, identified the microtubule-associated regulatory kinase, *Mark4*, as significantly up-regulated after focal axonal ischemia. Mark4 acts through site-specific phosphorylation of tau to destabilize microtubules [11] and is found in association with neurofibrillary tangles in AD brain [30]. Using a FRET-biosensor assay, we demonstrate that human Mark4 can potentiate tau aggregation in vitro. By associating subcortical ischemic axonal injury with molecular events occurring within connected cortical neurons, this study presents a strategy to identify novel molecular links between the two most common forms of dementia.

## Material and methods

### Animals

All animal studies presented here were approved by the UCLA Animal Research Committee, accredited by the AAALAC. Mice were housed under UCLA regulation with a 12-h dark-light cycle. All mice used in the study were male. Wild-type C57Bl/6 mice (Jackson Labs, Strain #000667) were used for all experiments unless otherwise stated. Male YFP-H transgenic mice (derived from B6.Cg-Tg (Thy1-YFP)HJrs/J mice, Jackson Labs, Strain #003782) were used for tissue clearing studies.

### White matter stroke

Subcortical white matter ischemic injury was induced as previously described [19, 37] using three focal injections of L-N^5^-(1-Iminoethyl) ornithine, Dihydrochloride (LNiO) added 1:1 (27 mg/mL, Millipore) with 20% fluororuby (Fluorochrome LLC) into white matter beneath sensorimotor cortex. Sham animals underwent fluororuby injections diluted in saline. Animals were sacrificed at 7 days post-stroke and either freshly dissected, fresh frozen on dry ice, or transcardially perfused with 4% PFA and prepared for tissue sections as described [19].

### Layer 5 MACS-FACS

At 1 week after stroke, regions of cortex overlying subcortical white matter stroke lesions were dissected and mechanically digested, following a single-cell suspension protocol using Neurocult dissociation kit (STEMCELL). For magnetic bead cell sorting (MACS), Neuronal enrichment kit microbeads and CD11b microbeads (Miltenyi Biotec) were added to the suspension before applying to MACS column to remove non-neuronal cells. After collecting all neuronal cells, we fixed and labeled neurons with Rabbit anti-CTIP2 (Abcam) (adapted from [34]) followed by anti-rabbit Alexa 488 and sorted for fluororuby and CTIP2+/Alexa488+ neurons at the Flow Cell Cytometry Core at UCLA. Total RNA was collected from sorted cells using NucleoSpin miRNA kit (Machery Nagel).

### RNA-seq

Isolated RNA from MACS-FACS isolated neurons was normalized by FACS cell counts to 10–20 cells/μL. cDNA library generation was performed using SMART-Seq v4 Ultra Low Input RNA Kit (Takara, Inc. #634894) and library products validated. RNA-sequencing was performed using TrueSeq with RiboZero (Illumina, Inc.) treatment. Samples were pooled, barcoded, and sequenced on an Illumina HiSeq 4000 sequencer over three RapidRun using 64 bp single end reads for an average of 34.9 M reads/sample. Reads were aligned to the latest mouse mm10 reference genome using the STAR spliced read aligner. Total counts of read-fragments aligned to known gene regions within the mouse mm10 refSeq reference annotation are used as the basis for quantification of gene expression. Fragment counts were derived using HTS-seq program. Various QC analyses were conducted to assess the quality of the data and to identify potential outliers. After exclusion of outlier samples, differential gene expression analysis was performed using limma-voom in R using a false discovery rate of < 0.1.

### Laser-capture microscopy

Twenty μm cryostat sections from fresh frozen specimens of sham and stroke animals were mounted on Arcturus Polyethylene Naphthalate (PEN) Membrane glass slides (Applied Biosystems, Inc.), briefly air-dried, fixed for 30 s in 95% EtOH. Sections from sham animals submerged in Cresyl Violet/EosinY solution [7] to identify Layer 5 cortical neurons by nuclear size, while sections from stroke animals were briefly rinsed in PBS. After sequential dehydration in ethanol and xylene, sections were stored in xylene until laser capture. Using a Leica Laser Microdissection (LMD) 7000 microscope, 20–50 Layer 5 cells per section were collected into RNA lysis buffer. RNA was then isolated as above.

### Confirmation of Mark4 up-regulation in cortical neurons

Laser capture microdissected neuronal RNA concentration was measured to create 2.5 ng/uL dilutions of total RNA from each sample and control (*n* = 4/grp). First-strand cDNA was created using SuperScript IV VILO Master Mix kit with ezDNase enzyme (Invitrogen). Targeted amplification of Mark4 and Gapdh was performed using Taqman PreAmp Master Mix kit (Thermo Fisher Scientific; Probe #Mm05549375_m1 (Mark4) and Mm99999915_g1 (Gapdh)) and relative gene expression determined using Taqman Gene Expression Master Mix (Thermo Fisher Scientific).

To determine Mark4 protein expression, 60-100X confocal fields of view were obtained from ipsilateral cortex overlying white matter stroke. Images were converted to 8-bit gray scale in Fiji [42] and the threshold equalized between all images. Total fluororuby cell number and Mark4-immunoreactive cell numbers were determined using the Cell counter plugin. To determine relative expression of Mark4, each Mark4 neuronal cell body was isolated and the mean arbitrary fluorescent units (AFUs) measured. Mark4 AFUs per unit area were averaged per animal to generate a relative expression level of Mark4 in FR+ and FR- cells.

### Immunofluorescence and confocal imaging

Fluororuby-labeled sham and stroke-injured brains were sectioned at 40 μm in a -22C cryostat and then stored in cryoprotectant at -20C. The total number of CTIP2+ and FR+ cells in the overlying motor and sensory cortices were measured using stereology. Briefly, regions of interest were outlined in Stereo Investigator (MBF Bioscience, Inc.) and positive cells counted using the optical fractionator. Total cell numbers were measured in 7–9 sections through the region of cortex overlying stroke. The ratio of FR+/CTIP2+ cells was generated for both sham (*n* = 3) and stroke-injured animals (*n* = 4). For staining, tissue sections containing stroke were removed from cryoprotectant and washed in PBS and incubated for 30 min in 10 mM sodium citrate buffer. After cooling and washing, sections were blocked in PBTDS and tissue was incubated overnight in either anti-MARK4 (Cell Signaling Cat# 4834S, 1:100), anti-phospho-tau Ser262 (Thermo Fisher Cat# 44–7506, 1:500), or 12e8 (gift from Benjamin Wolozin, 1:500) primary antibody in PBTDS. Corresponding secondary antibodies were added (1:250) including Donkey anti-Rabbit 488 or Donkey anti-Mouse 647 (Jackson ImmunoResearch) and counterstained with DAPI. Tissue was mounted onto glass slides and dehydrated in ethanol and xylenes and covered with DPX and a coverslip. Imaging was conducted on a Nikon C2 confocal microscope. Three 60X images were taken on each tissue section in the region of interest containing stroke-injured and non-stroke injured Layer 5 cortical neurons.

### U-DISCO & apical dendrite measurement

Seven days after white matter stroke in YFP-H transgenic mice (*n* = 5), animals were transcardially perfused with 4% PFA and post-fixed overnight at 4C. Tissue slabs 3 mm in thickness and spanning the region of stroke were generated that included left and right cortical regions. Tissues were cleared using uDISCO as described [38]. Briefly, tissues were optically cleared by serial incubation in increasing concentrations of tert-butanol (Acros Organics) followed by immersion in benzyl alcohol (Sigma-Aldrich)/benzyl benzoate (Sigma-Aldrich)/diphenyl ether (Alfa Aesar) (BABB-D) solution until transparent. The tissues were then immediately imaged on a Leica SP5 laser confocal microscope.

Apical dendrite length was measured in Fiji [42]. A standard grid was applied to images and neighboring YFP+ and YFP+/FR+ neurons (within 10 μm) were measured. The apical dendrite was measured by manual tracing beginning at the cell body and moving superiorly until the YFP signal was lost. Ten pairs of neurons were quantified per animal.

### Electro-chemiluminescence immunoassay (ECLIA) measurement of p-tau (Thr231)

Cortex overlying the white matter stroke was carefully isolated and dounce homogenized (20 times) in the presence of HALT Protease and Phosphatase inhibitor cocktail (Thermo Fisher Scientific, Waltham, MA) in PBS, and the total protein in each sample was quantified using BCA protein assay (Pierce) according to manufacturer instructions. The p-Tau (Thr231) concentration in cortex overlying stroke or sham was measured using a singleplex multi-spot phospho-Tau assay Kit (Meso Scale Discovery®, USA) as per the manufacturer’s recommendations. Briefly, blocker A was added to each well and incubated at room temperature with shaking at ~ 800 rpm for 1 hour. After washing the wells four times with the kit provided wash buffer, diluted samples and calibrators were added, and the plate was incubated at room temperature with shaking at ~ 800 rpm for 1 hour. After washing the wells four times with wash buffer, Sulfo-TAG detection antibody was added and the plate was incubated at room temperature with shaking at ~ 800 rpm for 1 hour. After washing thoroughly 150 μl of 1× read buffer were added, and the plate was read using an Meso Scale Discovery MESO QuickPlex SQ 120 instrument. The data were analyzed using Discover Workbench 4.0 software and quantified with reference to a freshly prepared standard curve.

### Immunoblotting

Dot blots were conducted using recombinant human Tau (Anaspec, #AS-55556) and recombinant human MARK4 (Abcam, #ab105211) and Mark/Par-1 inhibitor #39621 (Millipore Sigma, #454870). All recombinant proteins were dissolved in 25 mM Tris-HCl, pH 7.5. To measure tau phosphorylation recombinant human tau (15 μM) with or without recombinant human Mark4 (0.30 μM) were combined with the following: 5.0 mM ß-Glycerol phosphate; 12 mM MgCl_2_; 0.1 mM Na Orthovanadate; 2.0 mM Dithiothreitol; 50 μM ATP; 25 mM Tris-HCl, pH 7.5, and incubated at 30 °C. Four μL of each mixture were placed onto the membrane using a fine pipette tip. After all time points were collected, the membrane was blocked with 2.5% milk in TBS-T on an orbital shaker for 2 h at room temperature. The membrane was incubated overnight with the primary antibody to p-tau Ser262 (ThermoFisher Scientific, #OPA1–03142) diluted 1:1000 in 2.5% milk) at 4 °C. After three 5 min washes with TBS-T, the membrane was incubated for 1 h at room temperature with HRP-conjugated anti-Rb secondary antibody (Invitrogen, Cat#65–6120, diluted 1:10,000 in 2.5% milk). After three 5 min washes in TBS-T, the membrane was incubated with ECL reagent for 1 min and placed in a small plastic bag, and imaged with a Syngene PXi imager (MD).

### Cellular assay of tau aggregation using HEK biosensor cells

Tau RD P301S FRET Biosensor (ATCC CRL-3275) cells were cultured and analyzed as previously described [20]. The cells were grown in DMEM (Dulbecco’s modifications of eagle’s medium with 2 mM L-glutamine & 4.5G/L glucose) supplemented with fetal bovine serum 100 units/ml of penicillin G and 0.1 mg/ml of streptomycin sulfate in a humidified atmosphere of a 5% CO_2_ at 37 °C. Trypsinized-HEK293 cells were harvested, and seeded on collagen-coated 96-well flat plates (2.5–3.5 × 10^4^ cells/well) in 200 μl culture medium and incubated at 37 °C in 5% CO_2_ incubator. After 24 h, the prepared *Mark4* (1–250 pM) with or without Mark/Par-1 inhibitor #39621 (10 μM for 1 h) was pretreated with lipofectamine (Invitrogen) transfection reagent (0.2 μl/well). Tau repeat domain (residues 244–372), expressed as previously described [45], was aggregated until Thioflavin T fluorescence intensity reached a plateau, and was then diluted into Opti-MEM (GIBCO) and sonicated for 10 min in an ultrasonic water bath. After 48 h, the old culture media were replaced to fresh media and sonicated tau seeds with lipofectamine (Invitrogen) transfection reagent (0.2 μl/well) were treated. In experiments using mouse cortical homogenates or recombinant wild-type human tau, normalized amounts of total protein were determined using the BCA colorimetric assay, diluted in Opti-MEM and complexed with Lipofectamine 2000. Transduction complexes were incubated at room temperature for 20 min and then added directly to cells for 24–96 h. Tau aggregation of biosensor cells was visualized by florescent microscope images using FITC channel (ex: 485; em: 520) after 24 h. The cells were harvested after extensively washed and trypsinized. The harvested cells were moved in 200 μl chilled buffer (HBSS, 1% FBS, 1 mM EDTA), and then stored at 4 °C ready for FRET-based flow cytometry.

### Flow cytometry and data analysis of tau biosensor cells

FRET-based flow cytometry was used for quantifying the intracellular tau protein aggregation. The flow cytometry analyses of tau biosensor cells were performed by Digital Analyzers LSRII (IMED) flow cytometer. The FRET pair (ex: 405 nm; em: 525/50 nm) as well as CFP fusion protein (ex: 405 nm; em: 405/50 nm) and YFP fusion protein (ex: 488 nm; em: 525/50 nm) alone were measured for quatifying the fluorescence intensities. The FRET signal of the same amount of cells (20,000 cells per replicate) were analyzed for each experiment replicate to differentiate the aggregated tau protein from the non-aggregated status. The FRET gating was introduced to exclude all of the FRET-negative cells treated with PBS buffer and to include the FRET-positive cells treated with fibril seeds. The integrated FRET densities (IFD, FRET-positive cells multiplied by the median fluorescence intensity of FRET-positive cells) were calculated for all analyses. All flow cytometry data were analyzed to fit the non-linear sigmoidal curve. The quantified tau aggregation has conducted a minimum of three independent experiments with at least three replicates in each experimental condition.

### Statistical analysis

Data analysis was performed using Microsoft Excel, GraphPad Prism v7.0, and Matlab R2017a. Error bars shown in all graphs are standard error of the mean (SEM). Gene expression values were normalized and compared using a false-discovery rate adjusted *p*-value assuming significance at FDR < 0.1. Relative gene expression values generated by qPCR were compared using confidence intervals. A paired two-tailed t-test was used to compare the number of fluororuby and Mark4 cells. To determine differences in apical dendrite length, a Mann-Whitney two-tailed unpaired t-test was used to compare average Mark4 intensity per pixel per cell between co-labeled FR+/Mark+ and to FR−/Mark4+. ECLIA pTau levels were determined with a two-tailed unpaired t-test. Biosensor assay results were compared using a one-way ANOVA with Sidak’s multiple comparisons test. Unless otherwise stated, an α < 0.05 was used to determine statistical significance.

## Results

**Fig. 1:**
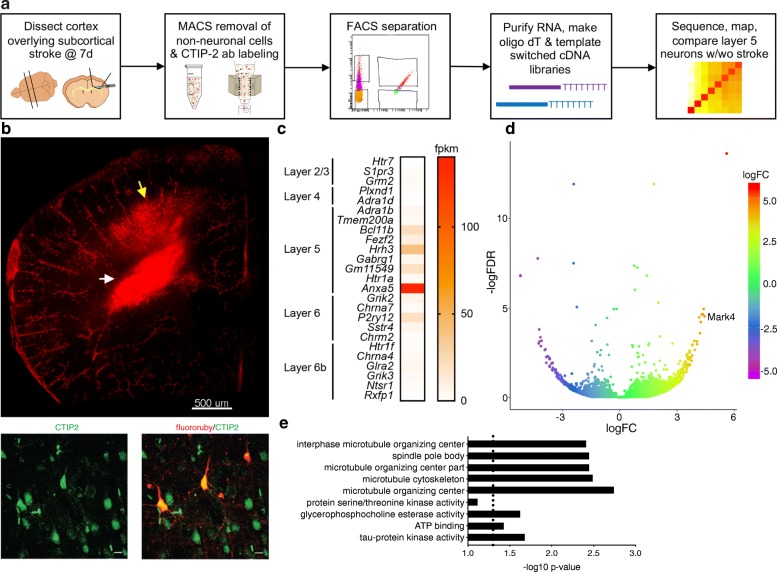
MACS-FACS-seq of Layer 5 cortical neurons after subcortical stroke. Schematic representation of workflow to isolate stroke-injured and neighboring uninjured CTIP2+ Layer 5 cortical neurons for RNA-sequencing (**a**). Retrograde neuronal tracing with fluororuby (FR) within subcortical white matter at the stroke site (white arrow, upper panel, **b**) and deep layers of overlying cortex (yellow arrow, upper panel, **b**) 7 days after stroke. CTIP2+ (green) and FR+ (red) Layer 5 cortical neurons overlying stroke (lower panels, **b**). Enrichment for Layer 5-specific marker genes in MACS-FACS isolated CTIP2+ cortical neurons (average fkpm; *n* = 5; *p* < 0.0001 by 2-way ANOVA) (**c**). Volcano plot of mapped gene sequences between CTIP2+/FR- and CTIP2+/FR+ cortical neurons (**d**). Gene ontology of differentially expressed genes (FDR < 0.1) (**e**). Dashed line indicates *p* = 0.05. Scale bars = 500 μm in upper panel and 10 μm in lower panels of **b**.

To determine the effect of subcortical ischemic axonal injury on cortical neurons, we used a workflow that allowed identification and RNA-sequencing of stroke-injured Layer 5 cortical neurons (Fig. 1a). We used a mouse model of focal ischemic white matter injury [19, 49] with retrograde tracer injections [48] to label sensorimotor cortical neurons with stroke-injured axons (Fig. 1b). This model provides a unique tool for identifying the effect of distal axonal injury on uninjured cortical neurons. Seven days after ischemic induction in wild-type mice, stroke-injured fluororuby+/CTIP2+ neurons (Fig. 1b, lower panels) within the overlying sensory and motor cortex were increased compared to sham (sensory: 0.26% ± 0.02 vs. 0.04% ± 0.005%; *p* < 0.0001; motor: 0.22% ± 0.01% vs. 0.07% ± 0.007%; *p* < 0.0001*)* (Additional file 1: Figure S1a-b) with subcortical stroke labeling an average of 0.24% ± 0.02**%** of the total CTIP2+ Layer 5 cortical neuron population in ipsilateral sensorimotor cortex overlying the ischemic lesions (Additional file 1: Figure S1c). To identify molecular programs specifically activated in stroke-injured CTIP2+ Layer 5 cortical neurons, we employed a magnetic-activated cell sorting (MACS)-fluorescence-activated cell sorting (FACS) method combined with CTIP2+ antibody labeling [34] followed by RNA-seq. MACS-FACS after stroke resulted in reliable detection of three cell populations with an average capture of 425.5 ± 157.9 FR+ cells, 45.5 ± 24.4 CTIP2+ cells, and 136.5 ± 43.2 FR+/CTIP2+ cells (*n* = 4) from each dissected cortical region (Additional file 1: Figure S2). After RNA isolation, we performed RNA-sequencing and paired sample differential gene expression between FR+/CTIP2+ stroke-injured neurons and neighboring CTIP2+ uninjured neurons. To verify that our layer-specific MACS-FACS-seq approach enriched for Layer 5 cortical neurons, we examined average fpkm for reported layer-specific cortical neuron marker genes [4] (Fig. 1c). This analysis confirmed enrichment of Layer 5 cortical neurons (F = 22.69, *p* < 0.0001 by one-way ANOVA) using CTIP2+ MACS-FACS-seq.

After controlling for outliers, differential gene expression analysis using limma-voom R package (FDR < 0.1) demonstrated 136 up-regulated and 85 down-regulated genes in Layer 5 cortical neurons 7d after subcortical ischemic axonal injury (Additional file 1: Figure S3; Additional file 2: Data File S1). Among the top up-regulated genes by logFC, was the microtubule affinity-regulating kinase *Mark4* (4.44-fold increased, FDR *p-value* = 2.96 × 10^− 5^) (Fig. 1d). The MARK family of enzymes play a key role in regulation of the cellular cytoskeleton [32]. In humans and rodents, there are four MARK isoforms, all of which have been implicated in AD and are found in association with hyperphosphorylated tau present in neurofibrillary tangles (NFTs) [30]. Among the MARK enzyme isoforms, only *Mark4* was enriched in stroke-injured cortical neurons. Gene ontology analysis [24] of the significantly up-regulated genes (FDR < 0.1) pointed to microtubule and cytoskeleton reorganization, including tau-protein kinase activity, as a key response of cortical neurons to subcortical stroke (Fig. 1e), further implicating *Mark4* as an axonal-ischemia response gene.

**Fig. 2:**
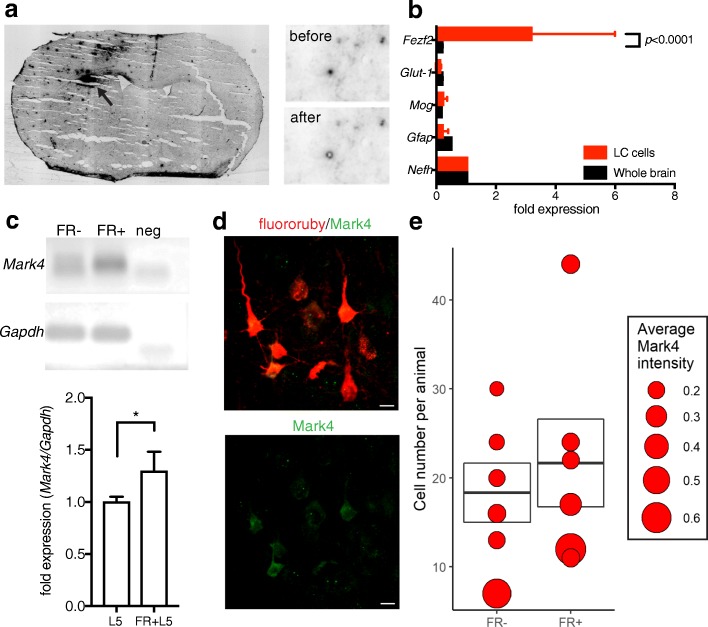
Mark4 up-regulation after subcortical stroke in Layer 5 cortical neurons. Image of fluororuby+ labeling in ipsilateral cortex after stroke (arrow) in sections prepared for laser capture microdissection (left, **a**). Individual fluororuby+ cortical neurons before (upper panel) and after (lower panel) laser capture microdissection (right, **a**). Graph of qPCR fold expression differences normalized to neurofilament heavy chain (NFH) for cell-type specific marker genes from laser-capture RNA (red) compared to whole brain RNA (black) (**b**) (*p* < 0.0001 by one-way ANOVA with adjusted *p*-value for *Fezf2* levels < 0.0001 by Sidak’s correction). Agarose gel of Mark4 PCR product from pooled LCM isolates (lower panel, **c**). qPCR for Mark4 in laser-captured FR- (L5) or FR+ (FR + L5) Layer 5 cortical neurons (1.3-fold increased expression, confidence interval ± 0.18, *n* = 4 in L5, *n* = 8 in FR + L5) (upper panel, **c**). Mark4 protein expression (green) in fluororuby+ (red) stroke-injured cortical neurons 7 days after stroke (**d**). Number of Mark4+ cells (bar plots with quartiles) and average intensity of Mark4 expression (AFUs/unit area) in FR- and FR+ cortical neurons after stroke (**e**) (*n* = 12, total cells = 240, *p* = 0.0053 for average Mark4 AFUs per animal by t-test). Mean ± S.E.M. Scale bars = 10 μm

To confirm *Mark4* up-regulation in cortical neurons after axonal ischemia, we performed laser capture microdissection of ipsilateral retrograde-labeled Layer 5 neurons 7d after stroke (453.5 ± 41.3 cells/animal*, n =* 8) compared to non-stroke injured Layer 5 neurons (455.8 ± 28.2 cells/animal*, n =* 4) (Fig. 2a). Enrichment of neuronal and Layer 5 neuron gene expression was confirmed by qPCR for salient glial marker genes and the Layer 5 marker gene *Fezf2* (Fig. 2b). We confirmed that *Mark4* gene expression was significantly up-regulated by qPCR from laser-captured neuronal isolates compared to control layer 5 cortical neurons (Fig. 2c). Within the cortex, fluororuby+/Mark4+ cells (Fig. 2d) represented ~ 30**%** of the stroke-injured fluororuby+ cells and stroke-injured neurons showed increased levels of Mark4 protein expression (Fig. 2e; 0.247 ± 0.097 AFUs in FR- cells vs. 0.306 ± 0.101 AFUs in FR+ cells, *p* = 0.0053) indicating that Layer 5 cortical neurons respond to subcortical ischemic axonal injury by up-regulating *Mark4* to remodel the cytoskeleton.

**Fig. 3:**
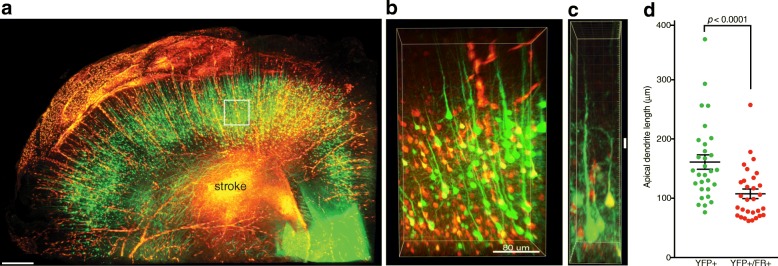
Reduction in dendritic complexity in Layer 5 cortical neurons after subcortical stroke. uDISCO cleared hemisphere of YFP-H+ transgenic mouse at 7 days after stroke with retrograde neuronal labeling using fluororuby (**a**). Stroke-injured FR+ (red) and neighboring uninjured YFP+ cortical neurons (green) are visible in sensorimotor cortex along with stroke-injured YFP+/FR+ neurons (yellow) (**b**). Apical dendrite length was measured in pairs of neighboring YFP+/FR- and YFP+/FR+ neurons (**c**). Graph of apical dendrite length in YFP+/FR+ cortical neurons compared to neighboring YFP+/FR- cortical neurons (**d**) (*n* = 3, total cells = 60, *p* < 0.0001 by paired t-test). Mean ± S.E.M. Scale bars = 300 μm in **a**, 80 μm in **b**, 10 μm in **c**

Overexpression of Mark4 in hippocampal neurons reduces their dendritic complexity [52]. To determine the consequence of Mark4 up-regulation in cortical neurons after subcortical axonal ischemia, we introduced subcortical strokes into the YFP-H transgenic line together with fluororuby [19]. We measured apical dendrite length and complexity in 3D using uDISCO (Fig. 3a-b; Additional file 3: Movie S1). As in wild-type mice, fluororuby labeling at the time of subcortical stroke results in robust neuronal labeling within the deep cortical layers including a significant fraction of YFP+ Layer 5 neurons. In YFP+/fluororuby+ stroke-injured neurons, apical dendrite length was reduced by 33.4% (161.0 ± 12.1 μm vs. 107.2 ± 8.0 μm; *p* < 0.0001 by paired t-test, *n* = 10 cells per animal per group*)* compared to neighboring but un-injured YFP+ neurons (Fig. 3c-d). These findings indicate that subcortical ischemic axonal injury reduces dendritic complexity in Layer 5 cortical neurons.

**Fig. 4:**
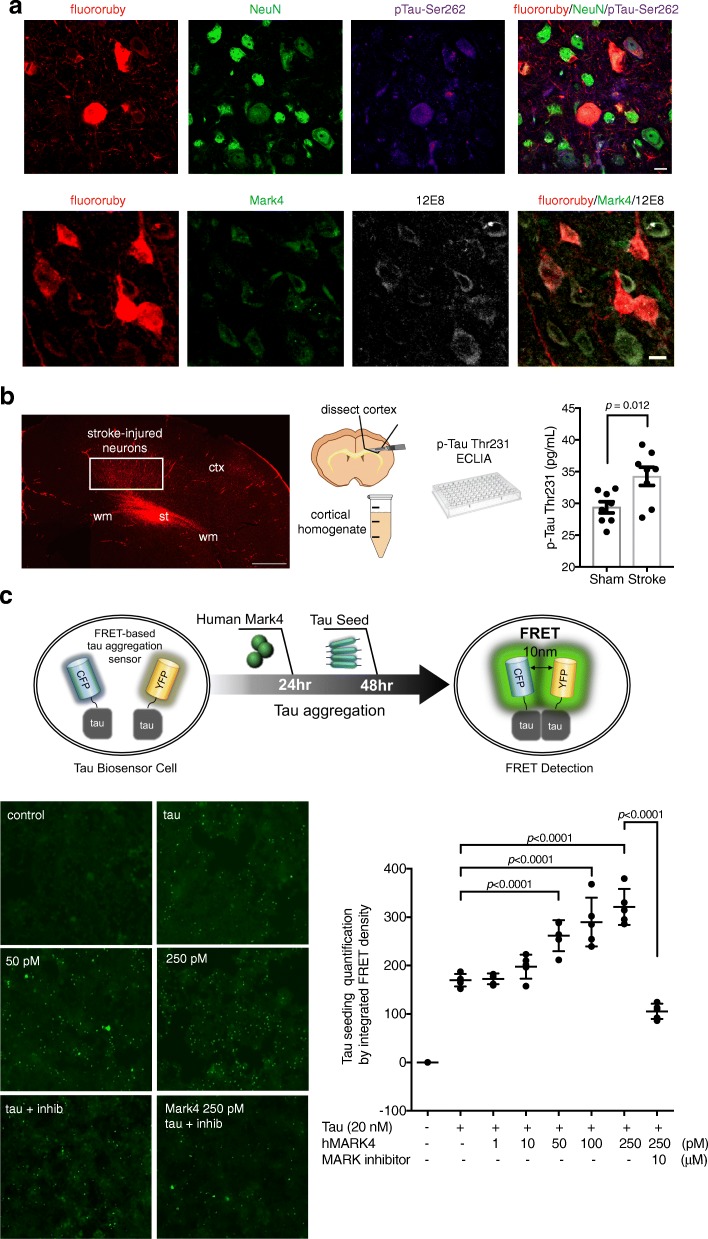
Mark4 potentiates tau phosphorylation in vivo and aggregation in vitro. Immunolabeling for pTau-Ser262 (purple) in stroke-injured FR+ (red) cells with uninjured NeuN+ cortical neurons (green) (upper panels, **a**). Immunolabeling for Mark4 (green) and 12E8 (white) in FR+ (red) cells (lower panels, **a**). Subcortical stroke with retrograde tracing highlighting stroke-injured cortical neurons 7d after stroke (left, **b**). Cortical tissue overlying stroke enriched for stroke-injured FR+ cells is selectively isolated (middle, **b**). ECLIA for pTau-Thr231 (pg/mL) in ipsilateral cortex of sham and stroke (**b**) (*n* = 8/grp, *p* = 0.012). Schematic of FRET-based tau biosensor assay used to measure tau aggregation in presence of human Mark4 (upper, **c**). Representative images of FRET signal induced by tau aggregation in presence of varying concentrations of transfected human Mark4 protein (pM) with or without Mark enzymatic inhibitor (left, **c**). Tau aggregation quantified by integrated FRET density in tau-biosensor cells in presence of 20 nM of tau repeat domains and increasing concentrations of human Mark4 (1–250 pM) and Mark enzymatic inhibition (10 μM) (right, **c**) (*p* < 0.0001 by ANOVA) with specific statistical comparison shown with brackets and *p-*values. Scale bars = 10 μm (**a**); 500 μm (**b**). Mean ± S.E.M

Mark4-mediated phosphorylation of Ser262 acts as a gate-keeper for subsequent, more pathogenic tau phosphorylation events [36]. To examine whether stroke-induced expression of Mark4 results in pathogenic tau phosphorylation, we examined murine tau phosphorylation after stroke using both immunofluorescence in stroke-injured neurons and electro-chemiluminescence immunoassay (ECLIA) for phospho-tau. Immunofluorescence labeling with a pTau-Ser262-specific antibody and the multi- epitope phosphoTau antibody 12E8 [29] demonstrated increased detection of phospho-tau in fluororuby positive cells, many of which were also Mark4+ (Fig. 4a). ECLIA for phospho-tau Thr231 levels showed an increase in tau phosphorylation in overlying cortical tissue from mice with subcortical ischemia compared to sham controls (*p =* 0.012, *n* = 8, Student’s t-test, Fig. 4b), indicating that ischemia damaging only the distal axonal projection of a cortical neuron is sufficient to promote pathogenic tau phosphorylation events.

The interaction between subcortical axonal ischemia and the up-regulation of a gene (*Mark4*) implicated in AD suggests a potential two-hit hypothesis for tauopathy related to dementia. To address this possibility, we used a FRET-based biosensor assay [20] to measure tau aggregation in HEK cells. Protein transfection of ipsilateral overlying cortical homogenates at 7d after stroke into tau biosensor cells did not result in significant tau aggregation by FRET (Additional file 1: Figure S4), nor did transfection of phosphorylated full-length recombinant human tau after pre-treatment with Mark4 (Additional file 1: Figure S4). However, the addition of human Mark4 protein into biosensor cells prior to seeding with the four-repeat domain of tau promotes the aggregation of tau in a both a dose-dependent (F = 72.22, *p* < 0.0001 by one-way ANOVA, Fig. 4c) and time-dependent manner (F = 159.3, *p <* 0.0001 by one-way ANOVA, Additional file 1: Figure S4). The Mark4-dependent increase in tau phosphorylation at Ser262 can be suppressed in vitro using a Mark-selective inhibitor (N-(2,5-Dimethylphenyl)-2-(4-(4-methoxyphenyl)-3-oxo-3,4-dihydropyrazin-2-ylthio)acetamide) [50] (Additional file 1: Figure S4). Addition of the inhibitor to Mark4-transfected biosensor cells similarly suppresses the effect of Mark4 on tau aggregation (adjusted *p* < 0.0001, Fig. 4c; Additional file 1: Figure S5). These results indicate that Mark4 potentiates tau aggregation and does so through phosphorylation at Ser262.

## Discussion

Cortical neurons have limited ability for repair after brain injury that leaves them vulnerable to degenerative phenomena. Recent advances in understanding molecular pathways in cortical neurons after stroke and traumatic brain injury have suggested that brain repair is possible if the molecular pathways regulating the response to injury are well-characterized [21, 28]. To date, models for understanding how neurons respond to selective axonal injury in the distal axon segments have been limited to the peripheral nerve system or the optic nerve using transection or crush injury models. Here, we illustrate how a stroke model of subcortical axonal ischemia together with retrograde neuronal tracing and cell capture can be used to identify molecular pathways that might be relevant for brain injury and repair in cortical neurons with isolated axonal damage. We chose to specifically isolate CTIP2+ Layer 5 cortical neurons since damage to subcortical axonal projections in this population accounts for motor dysfunction after stroke. Our layer-specific MACS-FACS-seq technique is internally controlled, enriches for the cell population of interest, and could be easily applied using other robust layer-specific markers. The use of slight fixation and labeling for intracellular markers does partially compromise RNA integrity but the application of RNA-sequencing advances in sequencing technology can partially compensate for this. We were also able to use cortical depth and size selection by laser-capture microdissection after subcortical axonal injury to identify the same population of cells, though LCM is more labor-intensive. Despite these challenges, we show definitive evidence that up-regulation of *Mark4* is a consistent response of cortical neurons to subcortical axonal ischemia.

Regulation of the neuronal cytoskeleton is a key response to injury after stroke [31]. Calcium-dependent pathways lead to turnover of synapses [23], dendritic restructuring [35], and remodeling of the axon initial segment in both directly damaged neurons [41] and those indirectly damaged by subcortical ischemia [19]. Here, we utilized uDISCO tissue clearing to show that cortical neurons undamaged by primary ischemia but subject to subcortical axonal ischemia undergo dendritic remodeling while unaffected neighboring neurons retain normal apical dendrite length. This cytoskeletal reorganization after subcortical axonal ischemia is consistent with the effects of Mark4 overexpression on dendritic complexity in cultured hippocampal neurons [52] indicating that this reorganization is at least partially dependent on the regulation of tau. Indeed, mice lacking *MAPT* subjected to hemispheric cerebral ischemia have reduced infarct volumes and preserved cognitive function [5] suggesting that post-stroke regulation of tau plays a central role in delayed neurodegeneration.

Mark4 has been implicated in the pathogenesis of AD through genetic linkage analysis [46], is found in association with NFTs in human AD brain, [30] and its principal phosphorylation site (serine 262) [17] within the tau repeat domains is thought to be critical to tau accumulation [10, 14]. Among the Mark enzyme isoforms (1–4), Mark4 is the most closely associated with Braak stage pathology in AD brain [17]. Its primary kinase activity is the phosphorylation of tau at Ser262 within the KXGS motif in the microtubule-binding domain of tau. Phosphorylation at Ser262 precedes the formation of NFTs [3] and can ultimately promote neuronal cell death [44]. The role of tau phosphorylation at Ser262 is controversial [9, 43] but the majority of evidence indicates that phosphorylation at Ser262 within the tau repeat domains acts as a gateway phosphorylation site that can promote additional phosphorylation events and can promote tau aggregation [6] and sensitize neurons to β-amyloid induced tau aggregation [1]. Here, we show that subcortical axonal ischemia not only induces phosphorylation at Ser262 but also promotes additional pathogenic tau phosphorylation events (Thr231). Because murine tau does not readily accumulate, further understanding of post-stroke modifications of tau in wild-type mice is limited. However, in a tau biosensor assay using pathogenic fragments of human tau, we were able to demonstrate that Mark4 potentiates tau aggregation and the inhibition of Mark enzymatic activity reduces rates of tau aggregation. This cell-based assay allows modeling of tau aggregation on a rapid time scale and our data demonstrate that in a cell with increased levels of Mark4, tau aggregation is promoted. The ability of stroke-injured neurons to handle these potentially pathogenic modifications of tau induced by Mark4 over the long-term is unknown.

Understanding the vascular contributions to Alzheimer’s disease (AD) is increasingly recognized as a critical step in developing the next generation of AD therapeutics [8]. Alzheimer’s disease and cerebrovascular disease account for over 80% of dementia diagnoses. The most common neuropathologic findings in vascular dementia are lacunar infarcts and microvascular ischemia in the brain white matter that are similar to the white matter lesions resulting from this stroke model [47]. At autopsy, at least half of patients with a clinical dementia diagnosis have mixed dementia, demonstrating hallmarks of chronic cerebrovascular disease in the form of subcortical ischemic white matter injury along with AD pathology [2]. In humans studies, white matter hyperintensities present on magnetic resonance imaging correlate with the degree of AD pathology in patients [13] and cerebrovascular pathology was significantly higher in a cohort of sporadic AD subjects compared to those with autosomal dominant AD [39]. The burden of cortical tau is also associated with white matter hyperintensities on MRI suggesting that white matter axonal injury is related to pathologic changes in the connected cortex [33]. Using in vivo PET tracers specific for aggregated tau (AV1451), Kim et al. [22] showed that increased tau accumulation was associated with the burden of cerebrovascular injury. These findings suggest an intriguing link between subcortical white matter ischemia and cortical tau accumulation. Here, we provide evidence that axonal ischemia triggers a molecular pathway that leads to the destabilization of tau from microtubules in deep cortical neurons. This molecular pathway may serve to link the two most common neurologic pathologies: subcortical white matter ischemic axonal injury and tauopathy associated with AD. Whether Mark4 is the sole enzymatic regulator of this cytoskeletal instability remains to be shown though testable by combining this white matter axonal injury model with appropriate AD transgenic mouse models. However, ischemia-induced priming of cortical neurons for pathogenic modifications of tau provides a novel drug target for mixed vascular and AD dementia. Given the commonality of both subcortical axonal ischemia *and* neurodegenerative pathologies in individuals with dementia, the contribution of axonal injury to pathways relevant to neurodegeneration deserves further investigation.

## Conclusions

In conclusion, these findings support a two-hit hypothesis for neurodegenerative disease in which ischemic axonal injury may function to prime cortical neurons for the pathologic changes associated with Alzheimer’s disease including neurofibrillary tangles. With its known role in regulating tau phosphorylation, Mark4 may serve a critical role in regulating the stability of the cytoskeleton in cortical neurons after subcortical injury making them more susceptible to tau accumulation. Though other up-stream signaling cascades induced by ischemic axonal injury may also contribute cytoskeletal remodeling, our in vitro biosensor assay findings suggest that Mark4 specifically potentiates pathogenic tau accumulation. Given the robust overlap of cerebrovascular and Alzheimer’s disease pathologies in the demented brain, the identification of other synergistic molecular pathways caused by stroke may lead to novel therapeutic targets for neurodegeneration.

## Additional files

Additional file 1: **Figure S1.** Retrograde neuronal labeling after subcortical stroke. **Figure S2.** MACS-FACS isolation of CTIP2+ Layer 5 cortical neurons after stroke. **Figure S3.** Gene expression differences in stroke-injured MACS-FACS isolated CTIP2+ neurons. **Figure S4.** Mark4 potentiates tau aggregation in a biosensor assay. **Figure S5.** Effect of Mark enzymatic inhibition on tau aggregation. (DOCX 25706 kb)

Additional file 2: **Data File S1.** Gene expression data from MACS-FACS isolated CTIP2+ Layer 5 cortical neurons. (XLSX 1172 kb)

Additional file 3: **Movie S1.** Tissue-cleared hemisphere from YFP-H transgenic mouse at 7 days after subcortical ischemic stroke with fluororuby neuronal tracing (red) to identify stroke-injured cortical neurons within sensorimotor cortex overlying a subcortical white matter stroke lesion. Neighboring uninjured (green) and stroke-injured (red) cortical neurons can be identified with a subpopulation of stroke-injured YFP+ neurons (yellow) that were used to measure changes in apical dendrite length after stroke. (MP4 8776 kb)

## Data Availability

The datasets generated during and/or analyzed during the current study are available from the corresponding author on reasonable request.
