# Supplementary material for: Ischemic axonal injury up-regulates MARK4 in cortical neurons and primes tau phosphorylation and aggregation
Source: Acta Neuropathol Commun. 2019 Aug 20;7:135. doi: 10.1186/s40478-019-0783-6 (PMC6700776; doi:10.1186/s40478-019-0783-6)
Supplement: Supplementary file 1 — Figure S1. Retrograde neuronal labeling after subcortical stroke. Figure S2. MACS-FACS isolation of CTIP2+ Layer 5 cortical neurons after stroke. Figure S3. Gene expression differences in stroke-injured MACS-FACS isolated CTIP2+ neurons. Figure S4. Mark4 potentiates tau aggregation in a biosensor assay. Figure S5. Effect of Mark enzymatic inhibition on tau aggregation. (DOCX 25706 kb) [file 40478_2019_783_MOESM1_ESM.docx]

Title: Ischemic axonal injury up-regulates MARK4 in cortical neurons and primes tau phosphorylation and aggregation

Authors: Eric Y. Hayden^1^, Jennifer Putman^1^, Stefanie Nunez^1^, Woo Shik Shin^1^, Mandavi Oberoi^1^, Malena Charreton^1^, Suman Dutta^1^, Zizheng Li^1^, Yutaro Komuro^1^, Mary Teena Joy^1^, Gal Bitan^1,2,3^, Allan MacKenzie-Graham^1^, Lin Jiang^1^, Jason D. Hinman^1^

**Supplemental Information**

**Supplemental Figures = 5**

**Figure S1. Retrograde neuronal labeling after subcortical stroke.** Fluororuby+ retrograde labeled cortical neurons in deep layers of sensorimotor cortex overlying stroke (a). Inset box shows single fluororuby+ neuron. Graph of average ratio of fluororuby+/CTIP2+ cortical neurons by stereology in 240 μm series in sham and stroke injured animals (*n*=3 in sham, *n*=4 in stroke) (bars represent group average, points represent group average per section overlying stroke injury; *p*<0.0001, t=8.467 for sensory cortex; *p*<0.0001, t=9.084 for motor cortex by unpaired t-test) (b). Graph of average ratio of fluororuby+/CTIP2+ cortical neurons between sham and stroke by anatomic position in overlying sensorimotor cortex through the region of stroke (*p*<0.0001 by two-way ANOVA for group difference, asterisks indicate unadjusted *p*-values for individual sections, *p*<0.05) (c). Error bars indicate S.E.M. Scale bar = 100 μm.

**Figure S2. MACS-FACS isolation of CTIP2+ Layer 5 cortical neurons after stroke.** Representative FACS plot showing post-MACS isolated populations of fluororuby+ (purple), CTIP2+ (green), and fluororuby+/CTIP2+ (red) neurons used for RNA-sequencing. Average number and range (± S.D.) of each isolated neuronal population is shown as a percentage of the total sort events (lower panel).

**Figure S3. Gene expression differences in stroke-injured MACS-FACS isolated CTIP2+ neurons.** Bar graph of up (red) and down (green)-regulated genes (FDR<0.1) between stroke-injured FR+/CTIP2+ and uninjured CTIP2+ neurons (a). Heatmap of Pearson correlation of variance stabilized transform of the raw expression counts for each paired population of MACS-FACS isolated neurons (b). Heatmap of differentially expressed genes in limma-voom with outliers removed. Red indicates up-regulated genes and green indicates down-regulated genes at *p*<0.005.

**Figure S4. Mark4 potentiates tau aggregation in a biosensor assay.** Dot blot for phospho-Tau-Ser262 after incubation of human Mark4 with human wild-type tau (left) and in presence of Mark inhibitor (right) (a) demonstrating the in vitro phosphorylation of tau by Mark4, and the inhibition of phosphorylation by a Mark inhibitor. Time-dependence of human Mark4 induction of tau aggregation in tau-biosensor cells using tau repeat domains as seeds (b) (p<0.0001 by two-way ANOVA, asterisks indicate adjusted p-values <0.0001 for comparisons between times), indicating that phosphorylation of tau increased the total amount of aggregation in this assay. Incubation of sham or post-stroke cortical tissue homogenate (mg) with tau-biosensor cells does not result in significant tau aggregation without tau repeat domain seeding showing that stroke-induced changes in mouse wild-type tau are not sufficient to induce aggregation (upper panels, c). Phosphorylation of human wild-type tau by Mark4 in vitro, with tau-biosensor cells does not result in significant tau aggregation without tau repeat domain seeding showing that full length human tau even when phosphorylated by Mark4 is not sufficient to induce aggregation (lower panels, c). Error bars indicate S.E.M.

**Figure S5. Effect of Mark enzymatic inhibition on tau aggregation.** Tau biosensor assay results in the presence or absence of increasing concentrations of the Mark-selective inhibitor (N-(2,5-Dimethylphenyl)-2-(4-(4-methoxyphenyl)-3-oxo-3,4-dihydropyrazin-2-ylthio)acetamide) (0.1-10 μM) with or without transfected human Mark4 protein (a). Representative images of FRET signal induced by tau aggregation in presence of varying concentrations of Mark inhibitor (0.1-10 μM; upper panels) and in the presence of Mark inhibitor (10 μM) and transfected human Mark4 protein (pM; lower panels) (b). Inhibition of Mark4 had no effect on tau aggregation in the absence of transfected Mark4 but reduced tau aggregation when Mark4 was abundant in the cell. There was no apparent cellular toxicity associated with exposure to Mark inhibitor. Error bars indicate S.E.M.
